# *t*-3-Pentyl-*r*-2,*c*-6-diphenyl­piperidin-4-one

**DOI:** 10.1107/S1600536809046753

**Published:** 2009-11-14

**Authors:** P. Gayathri, J. Jayabharathi, G. Rajarajan, A. Thiruvalluvar, R. J. Butcher

**Affiliations:** a PG Research Department of Physics, Rajah Serfoji Government College (Autonomous), Thanjavur 613 005, Tamilnadu, India; b Department of Chemistry, Annamalai University, Annamalai Nagar 608 002, Tamilnadu, India; c Department of Chemistry, Howard University, 525 College Street NW, Washington, DC 20059, USA

## Abstract

In the title mol­ecule, C_22_H_27_NO, the piperidine ring adopts a chair conformation, with all substituents equatorial. The dihedral angle between the two phenyl rings is 56.90 (5)°. In the crystal, mol­ecules are linked by weak C—H⋯O hydrogen bonds. A C—H⋯π inter­action involving the phenyl ring at the 6-position is also found in the crystal structure.

## Related literature

For a related crystal structure, see: Thiruvalluvar *et al.* (2007 ▶). For the biological activity ofpiperidines, see: Venketeshperumal *et al.* (2001 ▶).
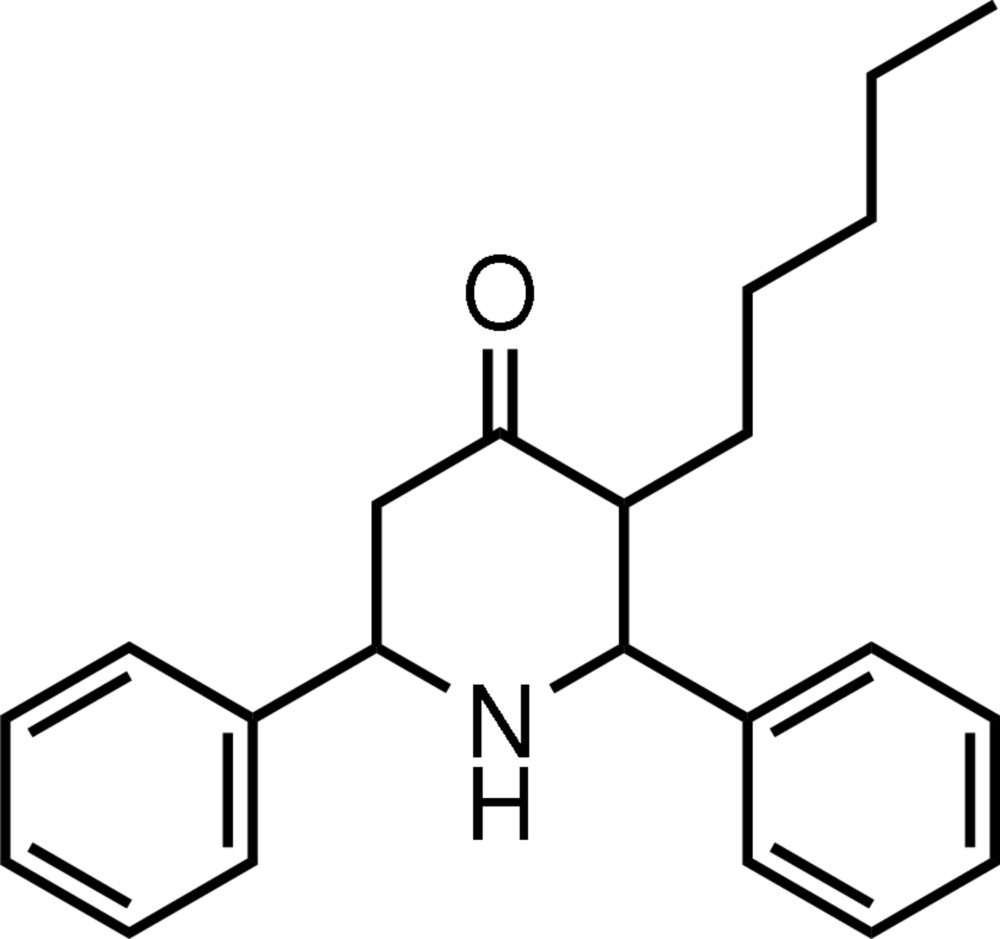


## Experimental

### 

#### Crystal data

C_22_H_27_NO
*M* *_r_* = 321.45
Monoclinic, 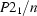

*a* = 12.2318 (5) Å
*b* = 5.5879 (2) Å
*c* = 26.9977 (10) Å
β = 94.377 (3)°
*V* = 1839.91 (12) Å^3^
*Z* = 4
Mo *K*α radiation
μ = 0.07 mm^−1^
*T* = 110 K
0.48 × 0.32 × 0.12 mm

#### Data collection

Oxford Diffraction Xcalibur Ruby Gemini diffractometer
Absorption correction: multi-scan (*CrysAlis Pro*; Oxford Diffraction, 2009 ▶) *T*_min_ = 0.937, *T*_max_ = 1.000
16194 measured reflections
6192 independent reflections
4118 reflections with *I* > 2σ(*I*)
*R*_int_ = 0.030

#### Refinement

*R*[*F* ^2^ > 2σ(*F* ^2^)] = 0.046
*wR*(*F* ^2^) = 0.121
*S* = 0.96
6192 reflections
221 parameters
H atoms treated by a mixture of independent and constrained refinement
Δρ_max_ = 0.31 e Å^−3^
Δρ_min_ = −0.18 e Å^−3^

### 

Data collection: *CrysAlis Pro* (Oxford Diffraction, 2009 ▶); cell refinement: *CrysAlis Pro*; data reduction: *CrysAlis Pro*; program(s) used to solve structure: *SHELXS97* (Sheldrick, 2008 ▶); program(s) used to refine structure: *SHELXL97* (Sheldrick, 2008 ▶); molecular graphics: *ORTEP-3* (Farrugia, 1997 ▶); software used to prepare material for publication: *PLATON* (Spek, 2009 ▶).

## Supplementary Material

Crystal structure: contains datablocks global, I. DOI: 10.1107/S1600536809046753/wn2365sup1.cif

Structure factors: contains datablocks I. DOI: 10.1107/S1600536809046753/wn2365Isup2.hkl

Additional supplementary materials:  crystallographic information; 3D view; checkCIF report

## Figures and Tables

**Table 1:** Hydrogen-bond geometry (Å, °)

*D*—H⋯*A*	*D*—H	H⋯*A*	*D*⋯*A*	*D*—H⋯*A*
C6—H6⋯O4^i^	1.00	2.59	3.2798 (11)	126
C34—H34*B*⋯*Cg*1^ii^	0.99	2.87	3.7809 (12)	154

Symmetry codes: (i) 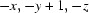; (ii) 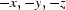. *Cg*1 is the centroid of the C61–C66 ring.

**Fig. 1.**
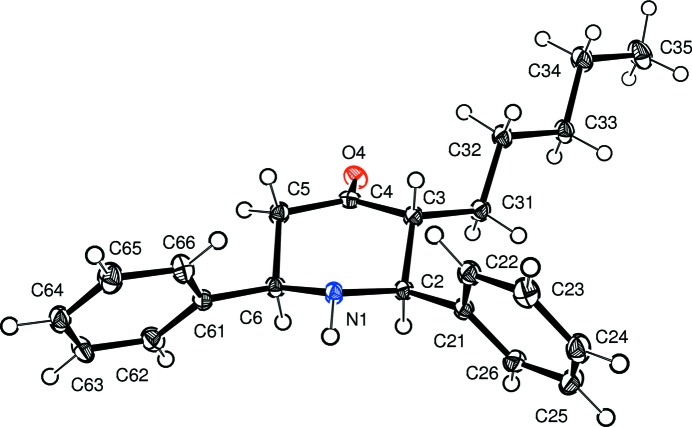
The molecular structure of the asymmetric unit, showing the atom-numbering scheme and displacement ellipsoids drawn at the 40% probability level. H atoms are shown as small spheres of arbitrary radius.

**Fig. 2.**
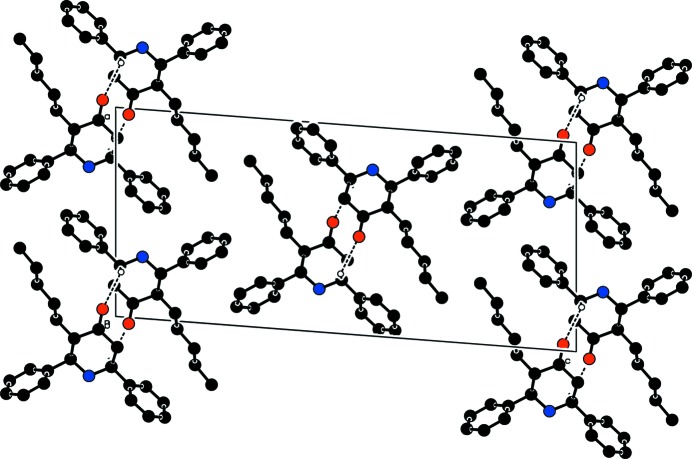
The packing of the title compound, viewed down the b axis. Dashed lines indicate C—H···O hydrogen bonds. H atoms not involved in hydrogen bonding have been omitted.

## supplementary crystallographic information

### Comment

Piperidones exhibit a wide spectrum of biological activities and form an essential part of the molecular structures of important drugs. Molecular geometry critically influences biological activity. Attention has been focused on structure-activity relationships. Piperidines with crowded groups at C3 and C5 have enhanced biological activity compared to other piperidines (Venketeshperumal *et al.*, 2001).

As part of our research, we have synthesized the title compound and report its crystal structure here. Thiruvalluvar *et al.*, (2007) have reported the crystal structure of a diphenylpiperidin-4-ol derivative, in which the piperidine ring adopts a chair conformation.

In the title molecule, C_22_H_27_NO, (Fig.1) the piperidine ring adopts a chair conformation, with all substituents equatorial. The dihedral angle between the two phenyl rings is 56.90 (5)°. Molecules are linked by C6—H6···O4(-*x*, 1 - *y*, -*z*) weak hydrogen bonds. A C34—H34B···π (-*x*, -*y*, -*z*) interaction involving the phenyl ring (C61—C66) is also found in the crystal structure.

### Experimental

A mixture of ammonium acetate (38.5 g, 0.5 mol), benzaldehyde (106.12 ml, 1 mol) and 2-octanone (64.10 ml, 0.5 mol) in distilled ethanol was heated to boiling. After cooling the viscous liquid obtained was dissolved in diethyl ether (200 ml) and shaken with 10 ml concentrated hydrochloric acid. The precipitated hydrochloride of the title compound was removed by filtration and washed with 40 ml mixture of ethanol and diethyl ether (1:1) and then with diethyl ether to remove most of the coloured impurities. The base was liberated from an alcoholic solution by adding aqueous ammonia and then diluting with water. It was purified by column chromatography, using an n-hexane-ethyl acetate mixture as the solvent. The yield of the compound was 80%.

### Refinement

The N-bound H atom was located in a difference Fourier map and refined freely; N1—H1 = 0.911 (12) Å. The remaining H atoms were positioned geometrically and allowed to ride on their parent atoms, with C—H = 0.95 - 1.00 Å; *U*_iso_(H) = k*U*_eq_(C), where k = 1.5 for methyl and 1.2 for all other H atoms.

### Crystal data

**Table:**

C_22_H_27_NO	*F*(000) = 696
*M**_r_* = 321.45	*D*_x_ = 1.160 Mg m^−^^3^
Monoclinic, *P*2_1_/*n*	Melting point: 368 K
Hall symbol: -P 2yn	Mo *K*α radiation, λ = 0.71073 Å
*a* = 12.2318 (5) Å	Cell parameters from 5712 reflections
*b* = 5.5879 (2) Å	θ = 4.9–32.7°
*c* = 26.9977 (10) Å	µ = 0.07 mm^−^^1^
β = 94.377 (3)°	*T* = 110 K
*V* = 1839.91 (12) Å^3^	Rectangular-plate, colourless
*Z* = 4	0.48 × 0.32 × 0.12 mm

### Data collection

**Table:**

Oxford Diffraction Xcalibur Ruby Gemini diffractometer	6192 independent reflections
Radiation source: Enhance (Mo) X-ray Source	4118 reflections with *I* > 2σ(*I*)
graphite	*R*_int_ = 0.030
Detector resolution: 10.5081 pixels mm^-1^	θ_max_ = 32.8°, θ_min_ = 5.0°
ω scans	*h* = −17→17
Absorption correction: multi-scan (*CrysAlis PRO*; Oxford Diffraction, 2009)	*k* = −8→8
*T*_min_ = 0.937, *T*_max_ = 1.000	*l* = −35→40
16194 measured reflections	

### Refinement

**Table:**

Refinement on *F*^2^	Primary atom site location: structure-invariant direct methods
Least-squares matrix: full	Secondary atom site location: difference Fourier map
*R*[*F*^2^ > 2σ(*F*^2^)] = 0.046	Hydrogen site location: inferred from neighbouring sites
*wR*(*F*^2^) = 0.121	H atoms treated by a mixture of independent and constrained refinement
*S* = 0.96	*w* = 1/[σ^2^(*F*_o_^2^) + (0.0674*P*)^2^] where *P* = (*F*_o_^2^ + 2*F*_c_^2^)/3
6192 reflections	(Δ/σ)_max_ = 0.001
221 parameters	Δρ_max_ = 0.31 e Å^−^^3^
0 restraints	Δρ_min_ = −0.18 e Å^−^^3^

### Special details

**Table:**

Geometry. Bond distances, angles *etc*. have been calculated using the rounded fractional coordinates. All su's are estimated from the variances of the (full) variance-covariance matrix. The cell e.s.d.'s are taken into account in the estimation of distances, angles and torsion angles
Refinement. Refinement of *F*^2^ against ALL reflections. The weighted *R*-factor *wR* and goodness of fit *S* are based on *F*^2^, conventional *R*-factors *R* are based on *F*, with *F* set to zero for negative *F*^2^. The threshold expression of *F*^2^ > 2σ(*F*^2^) is used only for calculating *R*-factors(gt) *etc*. and is not relevant to the choice of reflections for refinement. *R*-factors based on *F*^2^ are statistically about twice as large as those based on *F*, and *R*- factors based on ALL data will be even larger.

### Fractional atomic coordinates and isotropic or equivalent isotropic displacement parameters (Å^2^)

**Table:**

	*x*	*y*	*z*	*U*_iso_*/*U*_eq_	
O4	−0.02984 (5)	0.25856 (13)	0.02836 (2)	0.0227 (2)	
N1	0.29439 (6)	0.34289 (15)	0.05803 (3)	0.0171 (2)	
C2	0.22093 (7)	0.38195 (17)	0.09795 (3)	0.0165 (2)	
C3	0.12069 (7)	0.21283 (18)	0.09013 (3)	0.0169 (3)	
C4	0.06862 (8)	0.23999 (17)	0.03724 (3)	0.0173 (2)	
C5	0.14663 (8)	0.24418 (18)	−0.00331 (3)	0.0192 (3)	
C6	0.24286 (7)	0.41460 (18)	0.00937 (3)	0.0172 (3)	
C21	0.28492 (7)	0.34132 (18)	0.14743 (3)	0.0172 (3)	
C22	0.34717 (8)	0.13503 (19)	0.15574 (3)	0.0207 (3)	
C23	0.40971 (8)	0.1003 (2)	0.20020 (4)	0.0255 (3)	
C24	0.40946 (9)	0.2723 (2)	0.23750 (4)	0.0291 (3)	
C25	0.34617 (9)	0.4751 (2)	0.23009 (4)	0.0293 (3)	
C26	0.28428 (8)	0.51060 (19)	0.18526 (4)	0.0225 (3)	
C31	0.03717 (8)	0.24628 (18)	0.12901 (4)	0.0201 (3)	
C32	−0.03780 (8)	0.0314 (2)	0.13360 (4)	0.0221 (3)	
C33	−0.12480 (8)	0.06791 (19)	0.17050 (4)	0.0219 (3)	
C34	−0.20306 (9)	−0.1429 (2)	0.17217 (4)	0.0299 (3)	
C35	−0.29164 (9)	−0.1088 (3)	0.20818 (4)	0.0348 (4)	
C61	0.32404 (7)	0.40532 (18)	−0.03020 (3)	0.0173 (3)	
C62	0.32447 (9)	0.58540 (19)	−0.06565 (4)	0.0248 (3)	
C63	0.39528 (9)	0.5724 (2)	−0.10367 (4)	0.0294 (3)	
C64	0.46508 (8)	0.3801 (2)	−0.10661 (4)	0.0264 (3)	
C65	0.46548 (9)	0.1999 (2)	−0.07135 (4)	0.0276 (3)	
C66	0.39528 (8)	0.2136 (2)	−0.03327 (4)	0.0241 (3)	
H1	0.3572 (10)	0.428 (2)	0.0651 (4)	0.021 (3)*	
H2	0.19459	0.55139	0.09639	0.0197*	
H3	0.14887	0.04518	0.09352	0.0203*	
H5A	0.10677	0.29558	−0.03481	0.0230*	
H5B	0.17510	0.08071	−0.00819	0.0230*	
H6	0.21426	0.58156	0.01167	0.0206*	
H22	0.34686	0.01608	0.13059	0.0249*	
H23	0.45262	−0.04059	0.20520	0.0306*	
H24	0.45269	0.24994	0.26790	0.0349*	
H25	0.34479	0.59133	0.25572	0.0351*	
H26	0.24127	0.65141	0.18044	0.0270*	
H31A	0.07699	0.27722	0.16169	0.0242*	
H31B	−0.00827	0.38878	0.12012	0.0242*	
H32A	0.00745	−0.10925	0.14406	0.0266*	
H32B	−0.07477	−0.00435	0.10053	0.0266*	
H33A	−0.08807	0.09314	0.20405	0.0263*	
H33B	−0.16739	0.21403	0.16129	0.0263*	
H34A	−0.16033	−0.28830	0.18179	0.0359*	
H34B	−0.23859	−0.16958	0.13844	0.0359*	
H35A	−0.33880	−0.25068	0.20750	0.0522*	
H35B	−0.33584	0.03216	0.19838	0.0522*	
H35C	−0.25732	−0.08574	0.24184	0.0522*	
H62	0.27630	0.71802	−0.06399	0.0298*	
H63	0.39541	0.69675	−0.12767	0.0352*	
H64	0.51277	0.37121	−0.13272	0.0317*	
H65	0.51358	0.06723	−0.07319	0.0331*	
H66	0.39612	0.09009	−0.00905	0.0289*	

### Atomic displacement parameters (Å^2^)

**Table:**

	*U*^11^	*U*^22^	*U*^33^	*U*^12^	*U*^13^	*U*^23^
O4	0.0151 (3)	0.0264 (4)	0.0265 (4)	−0.0013 (3)	0.0007 (3)	0.0036 (3)
N1	0.0131 (4)	0.0236 (4)	0.0147 (3)	−0.0032 (3)	0.0021 (3)	−0.0007 (3)
C2	0.0157 (4)	0.0180 (4)	0.0160 (4)	−0.0013 (4)	0.0035 (3)	−0.0019 (3)
C3	0.0142 (4)	0.0196 (5)	0.0173 (4)	−0.0018 (4)	0.0031 (3)	−0.0017 (4)
C4	0.0157 (4)	0.0148 (4)	0.0213 (4)	−0.0026 (4)	0.0013 (3)	−0.0008 (3)
C5	0.0168 (4)	0.0238 (5)	0.0170 (4)	−0.0019 (4)	0.0012 (3)	−0.0021 (4)
C6	0.0160 (4)	0.0189 (5)	0.0168 (4)	0.0000 (4)	0.0018 (3)	0.0002 (3)
C21	0.0135 (4)	0.0226 (5)	0.0160 (4)	−0.0051 (4)	0.0038 (3)	−0.0017 (4)
C22	0.0178 (4)	0.0252 (5)	0.0196 (4)	−0.0017 (4)	0.0041 (3)	−0.0013 (4)
C23	0.0180 (5)	0.0330 (6)	0.0255 (5)	0.0004 (4)	0.0010 (4)	0.0041 (4)
C24	0.0237 (5)	0.0440 (7)	0.0192 (5)	−0.0096 (5)	−0.0015 (4)	0.0018 (5)
C25	0.0319 (6)	0.0363 (6)	0.0198 (5)	−0.0100 (5)	0.0025 (4)	−0.0072 (4)
C26	0.0232 (5)	0.0236 (5)	0.0211 (5)	−0.0044 (4)	0.0051 (4)	−0.0040 (4)
C31	0.0170 (4)	0.0247 (5)	0.0193 (4)	−0.0027 (4)	0.0053 (3)	−0.0037 (4)
C32	0.0209 (5)	0.0265 (5)	0.0197 (5)	−0.0039 (4)	0.0060 (4)	−0.0012 (4)
C33	0.0182 (4)	0.0283 (5)	0.0198 (4)	−0.0014 (4)	0.0050 (3)	0.0008 (4)
C34	0.0253 (5)	0.0393 (7)	0.0260 (5)	−0.0101 (5)	0.0072 (4)	−0.0004 (5)
C35	0.0249 (5)	0.0461 (8)	0.0347 (6)	−0.0062 (5)	0.0101 (5)	0.0083 (5)
C61	0.0150 (4)	0.0216 (5)	0.0153 (4)	−0.0044 (4)	0.0011 (3)	−0.0013 (3)
C62	0.0281 (5)	0.0227 (5)	0.0240 (5)	−0.0010 (4)	0.0044 (4)	0.0030 (4)
C63	0.0345 (6)	0.0324 (6)	0.0219 (5)	−0.0099 (5)	0.0071 (4)	0.0057 (4)
C64	0.0204 (5)	0.0401 (7)	0.0193 (5)	−0.0113 (5)	0.0056 (4)	−0.0045 (4)
C65	0.0208 (5)	0.0364 (6)	0.0263 (5)	0.0020 (5)	0.0066 (4)	−0.0025 (5)
C66	0.0238 (5)	0.0275 (5)	0.0216 (5)	0.0024 (4)	0.0059 (4)	0.0039 (4)

### Geometric parameters (Å, °)

**Table:**

O4—C4	1.2143 (12)	C2—H2	1.0000
N1—C2	1.4712 (11)	C3—H3	1.0000
N1—C6	1.4688 (12)	C5—H5A	0.9900
N1—H1	0.911 (12)	C5—H5B	0.9900
C2—C3	1.5497 (13)	C6—H6	1.0000
C2—C21	1.5130 (12)	C22—H22	0.9500
C3—C31	1.5310 (13)	C23—H23	0.9500
C3—C4	1.5260 (12)	C24—H24	0.9500
C4—C5	1.5063 (13)	C25—H25	0.9500
C5—C6	1.5323 (13)	C26—H26	0.9500
C6—C61	1.5139 (12)	C31—H31A	0.9900
C21—C22	1.3903 (14)	C31—H31B	0.9900
C21—C26	1.3925 (14)	C32—H32A	0.9900
C22—C23	1.3869 (14)	C32—H32B	0.9900
C23—C24	1.3922 (16)	C33—H33A	0.9900
C24—C25	1.3784 (16)	C33—H33B	0.9900
C25—C26	1.3919 (15)	C34—H34A	0.9900
C31—C32	1.5216 (15)	C34—H34B	0.9900
C32—C33	1.5259 (15)	C35—H35A	0.9800
C33—C34	1.5207 (15)	C35—H35B	0.9800
C34—C35	1.5219 (16)	C35—H35C	0.9800
C61—C66	1.3874 (14)	C62—H62	0.9500
C61—C62	1.3890 (14)	C63—H63	0.9500
C62—C63	1.3946 (15)	C64—H64	0.9500
C63—C64	1.3785 (15)	C65—H65	0.9500
C64—C65	1.3854 (16)	C66—H66	0.9500
C65—C66	1.3910 (15)		
			
O4···C32	3.1202 (12)	H5A···O4^i^	2.6700
O4···C6^i^	3.2798 (11)	H5A···H32B^ii^	2.4200
O4···C4^i^	3.3294 (11)	H5B···C66	2.9200
O4···O4^i^	3.2139 (10)	H5B···O4^ii^	2.6300
O4···C4^ii^	3.3158 (11)	H6···H2	2.3200
O4···C5^ii^	3.2004 (12)	H6···H62	2.3600
O4···C5^i^	3.1736 (12)	H6···O4^i^	2.5900
O4···H32B	2.5300	H22···N1	2.7200
O4···H31B	2.5800	H22···C3	3.1000
O4···H5A^i^	2.6700	H22···H3	2.5500
O4···H5B^ii^	2.6300	H22···H65^ix^	2.4400
O4···H6^i^	2.5900	H23···C25^vi^	3.1000
N1···H22	2.7200	H24···H32A^iv^	2.5200
N1···H66	2.6800	H25···H31A^iv^	2.5800
C4···O4^i^	3.3294 (11)	H26···C22^vii^	3.0900
C4···O4^ii^	3.3158 (11)	H26···H2	2.3600
C5···O4^ii^	3.2004 (12)	H26···C24^iv^	3.0600
C5···O4^i^	3.1736 (12)	H31A···C21	2.6300
C6···O4^i^	3.2798 (11)	H31A···C26	2.8800
C24···C26^iii^	3.5857 (15)	H31A···H25^iii^	2.5800
C26···C31	3.5949 (14)	H31B···O4	2.5800
C26···C24^iv^	3.5857 (15)	H31B···H33B	2.5100
C31···C26	3.5949 (14)	H32A···H3	2.4400
C32···O4	3.1202 (12)	H32A···H34A	2.5600
C3···H22	3.1000	H32A···H24^iii^	2.5200
C4···H32B	2.8800	H32B···O4	2.5300
C5···H32B^ii^	3.0200	H32B···C4	2.8800
C21···H64^v^	3.0000	H32B···H34B	2.5000
C21···H31A	2.6300	H32B···C5^ii^	3.0200
C22···H26^vi^	3.0900	H32B···H5A^ii^	2.4200
C22···H3	2.8900	H33A···H35C	2.5800
C22···H1	2.955 (11)	H33B···H31B	2.5100
C24···H26^iii^	3.0600	H33B···H35B	2.5700
C25···H23^vii^	3.1000	H34A···H32A	2.5600
C26···H64^v^	3.0200	H34B···H32B	2.5000
C26···H31A	2.8800	H34B···C63^ii^	3.0600
C35···H35C^viii^	3.0300	H34B···C64^ii^	3.0700
C63···H34B^ii^	3.0600	H35A···H35C^viii^	2.5500
C64···H1^v^	2.600 (12)	H35B···H33B	2.5700
C64···H34B^ii^	3.0700	H35B···H63^i^	2.5000
C65···H1^v^	3.000 (12)	H35C···H33A	2.5800
C66···H1	2.982 (11)	H35C···C35^x^	3.0300
C66···H5B	2.9200	H35C···H35A^x^	2.5500
H1···C22	2.955 (11)	H62···H6	2.3600
H1···C66	2.982 (11)	H63···H35B^i^	2.5000
H1···C64^v^	2.600 (12)	H64···C21^v^	3.0000
H1···C65^v^	3.000 (12)	H64···C26^v^	3.0200
H1···H64^v^	2.5800	H64···H1^v^	2.5800
H2···H6	2.3200	H65···H22^ix^	2.4400
H2···H26	2.3600	H65···H66^ix^	2.5600
H3···C22	2.8900	H66···N1	2.6800
H3···H22	2.5500	H66···H65^ix^	2.5600
H3···H32A	2.4400		
			
C2—N1—C6	111.74 (7)	C5—C6—H6	109.00
C6—N1—H1	110.0 (7)	C61—C6—H6	109.00
C2—N1—H1	108.8 (7)	C21—C22—H22	120.00
N1—C2—C21	108.71 (7)	C23—C22—H22	120.00
N1—C2—C3	109.32 (7)	C22—C23—H23	120.00
C3—C2—C21	112.30 (7)	C24—C23—H23	120.00
C4—C3—C31	112.13 (8)	C23—C24—H24	120.00
C2—C3—C4	109.65 (7)	C25—C24—H24	120.00
C2—C3—C31	113.24 (7)	C24—C25—H25	120.00
O4—C4—C5	121.93 (7)	C26—C25—H25	120.00
O4—C4—C3	122.01 (8)	C21—C26—H26	120.00
C3—C4—C5	116.06 (8)	C25—C26—H26	120.00
C4—C5—C6	111.45 (7)	C3—C31—H31A	109.00
N1—C6—C5	107.47 (7)	C3—C31—H31B	109.00
C5—C6—C61	110.80 (7)	C32—C31—H31A	109.00
N1—C6—C61	111.22 (7)	C32—C31—H31B	109.00
C2—C21—C26	120.94 (9)	H31A—C31—H31B	108.00
C2—C21—C22	120.46 (8)	C31—C32—H32A	109.00
C22—C21—C26	118.59 (8)	C31—C32—H32B	109.00
C21—C22—C23	120.99 (9)	C33—C32—H32A	109.00
C22—C23—C24	119.86 (10)	C33—C32—H32B	109.00
C23—C24—C25	119.66 (10)	H32A—C32—H32B	108.00
C24—C25—C26	120.37 (10)	C32—C33—H33A	109.00
C21—C26—C25	120.52 (9)	C32—C33—H33B	109.00
C3—C31—C32	113.41 (8)	C34—C33—H33A	109.00
C31—C32—C33	113.72 (9)	C34—C33—H33B	109.00
C32—C33—C34	112.81 (9)	H33A—C33—H33B	108.00
C33—C34—C35	113.73 (10)	C33—C34—H34A	109.00
C6—C61—C62	119.91 (9)	C33—C34—H34B	109.00
C62—C61—C66	118.87 (9)	C35—C34—H34A	109.00
C6—C61—C66	121.16 (9)	C35—C34—H34B	109.00
C61—C62—C63	120.31 (10)	H34A—C34—H34B	108.00
C62—C63—C64	120.34 (10)	C34—C35—H35A	109.00
C63—C64—C65	119.78 (10)	C34—C35—H35B	109.00
C64—C65—C66	119.86 (10)	C34—C35—H35C	109.00
C61—C66—C65	120.84 (10)	H35A—C35—H35B	109.00
N1—C2—H2	109.00	H35A—C35—H35C	109.00
C3—C2—H2	109.00	H35B—C35—H35C	109.00
C21—C2—H2	109.00	C61—C62—H62	120.00
C2—C3—H3	107.00	C63—C62—H62	120.00
C4—C3—H3	107.00	C62—C63—H63	120.00
C31—C3—H3	107.00	C64—C63—H63	120.00
C4—C5—H5A	109.00	C63—C64—H64	120.00
C4—C5—H5B	109.00	C65—C64—H64	120.00
C6—C5—H5A	109.00	C64—C65—H65	120.00
C6—C5—H5B	109.00	C66—C65—H65	120.00
H5A—C5—H5B	108.00	C61—C66—H66	120.00
N1—C6—H6	109.00	C65—C66—H66	120.00
			
C6—N1—C2—C3	65.82 (9)	N1—C6—C61—C66	44.07 (12)
C6—N1—C2—C21	−171.28 (8)	C5—C6—C61—C62	101.57 (10)
C2—N1—C6—C5	−66.24 (9)	C5—C6—C61—C66	−75.40 (11)
C2—N1—C6—C61	172.32 (8)	C2—C21—C22—C23	177.34 (9)
N1—C2—C3—C4	−51.99 (10)	C26—C21—C22—C23	−1.64 (14)
N1—C2—C3—C31	−178.04 (8)	C2—C21—C26—C25	−177.96 (9)
C21—C2—C3—C4	−172.73 (7)	C22—C21—C26—C25	1.02 (14)
C21—C2—C3—C31	61.21 (10)	C21—C22—C23—C24	0.85 (15)
N1—C2—C21—C22	−49.98 (11)	C22—C23—C24—C25	0.58 (16)
N1—C2—C21—C26	128.98 (9)	C23—C24—C25—C26	−1.19 (16)
C3—C2—C21—C22	71.12 (11)	C24—C25—C26—C21	0.38 (16)
C3—C2—C21—C26	−109.92 (10)	C3—C31—C32—C33	−177.25 (8)
C2—C3—C4—O4	−134.43 (9)	C31—C32—C33—C34	176.50 (9)
C2—C3—C4—C5	45.15 (11)	C32—C33—C34—C35	−179.13 (9)
C31—C3—C4—O4	−7.74 (13)	C6—C61—C62—C63	−176.86 (9)
C31—C3—C4—C5	171.84 (8)	C66—C61—C62—C63	0.17 (15)
C2—C3—C31—C32	−159.31 (8)	C6—C61—C66—C65	176.46 (9)
C4—C3—C31—C32	75.97 (11)	C62—C61—C66—C65	−0.54 (15)
O4—C4—C5—C6	132.23 (9)	C61—C62—C63—C64	0.37 (16)
C3—C4—C5—C6	−47.35 (11)	C62—C63—C64—C65	−0.55 (16)
C4—C5—C6—N1	54.81 (10)	C63—C64—C65—C66	0.18 (16)
C4—C5—C6—C61	176.51 (8)	C64—C65—C66—C61	0.37 (16)
N1—C6—C61—C62	−138.96 (9)		

Symmetry codes: (i) −*x*, −*y*+1, −*z*; (ii) −*x*, −*y*, −*z*; (iii) −*x*+1/2, *y*−1/2, −*z*+1/2; (iv) −*x*+1/2, *y*+1/2, −*z*+1/2; (v) −*x*+1, −*y*+1, −*z*; (vi) *x*, *y*−1, *z*; (vii) *x*, *y*+1, *z*; (viii) −*x*−1/2, *y*−1/2, −*z*+1/2; (ix) −*x*+1, −*y*, −*z*; (x) −*x*−1/2, *y*+1/2, −*z*+1/2.

### Hydrogen-bond geometry (Å, °)

**Table:**

*D*—H···*A*	*D*—H	H···*A*	*D*···*A*	*D*—H···*A*
C6—H6···O4^i^	1.00	2.59	3.2798 (11)	126
C34—H34B···Cg1^ii^	0.99	2.87	3.7809 (12)	154

Symmetry codes: (i) −*x*, −*y*+1, −*z*; (ii) −*x*, −*y*, −*z*.
